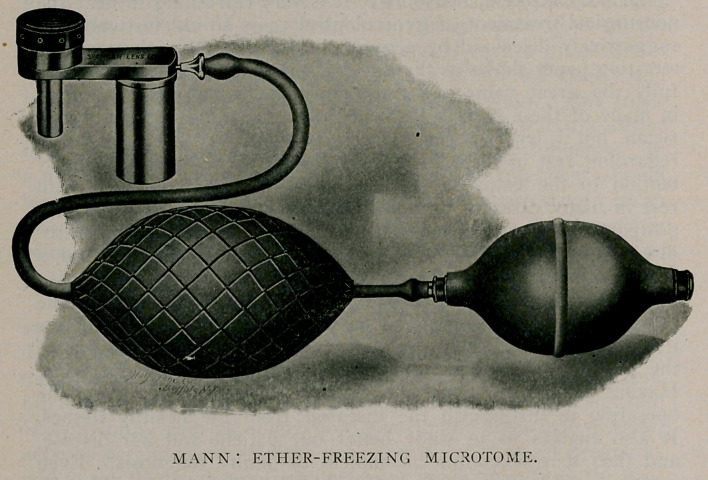# A Compact Ether-Freezing Microtome

**Published:** 1908-10

**Authors:** Edward C. Mann

**Affiliations:** Medical Department, University of Buffalo


### A Compact Ether-Freezing Microtome.

By EDWARD C. MANN., M. D.
Medical Department, University of Buffalo.

THE accompanying cut shows an ether-freezing apparatus which can be used with any microtome and by which the general practitioner is enabled to make a quick diagnosis.

Numerous methods of freezing material for microscopical sectioning have been used, chief among which are the carbon dioxid method, salt and ice, and ether freezing by means of a spray. The carbon dioxid, although perhaps the best method for a large laboratory, freezing larger pieces of material, is impracticable for small laboratories and the general practitioner. The tanks of carbon dioxid are expensive and apt to leak. The apparatus is quite expensive and cannot be obtained easily in all places. The tanks of gas are often found empty just when one wants to use them, and not rarely they contain a good deal of water and iron rust.

MANN: ETHER-FREEZING MICROTOME.

The salt and ice method gives much more trouble, is slower and not so cleanly as the method here shown. A physician always has ether in his office. Ether freezing is then the ideal method if an apparatus which is not cumbersome, will not wear, and which will freeze the material can be had. In particular most ether freezing atomisers require rubber tubing for the connections. The rubber dissolves and soon plugs the fine tubes. The cut shows a convenient and compact ether atomiser constructed entirely of metal, devised by the writer. The ether is sprayed in finely divided globules by means of an ordinary atomiser nozzle. That which does not evaporate is run off from the spraying part into the cup which holds about 10 cc. of ether, enough to freeze a piece of material 1 m. m. thick and 1 cm. square, thus allowing sufficient number of sections to be cut with a minimum of ether and time. The material should be fixed in a 10 per cent. formalin solution, that is, (10 per cent. of the 40 per cent. solution of formaldehyde) as is usual in all methods. Certain forms of tissue, as fat, are very hard to freeze, but for general work and quick diagnosis, the apparatus here shown, made by the Spencer Lens Company, will be found very satisfactory. Only the best quality of ether should be used.

37 Allen Street.

## Figures and Tables

**Figure f1:**